# Predictors of cervical cancer screening intention of HIV-positive women in the central region of Ghana

**DOI:** 10.1186/s12905-018-0534-z

**Published:** 2018-02-27

**Authors:** Nancy Innocentia Ebu, Joseph Kwesi Ogah

**Affiliations:** 1 Department of Public Health, School of Nursing and Midwifery, University of Cape Coast, Cape Coast, Ghana; 2 Department of Health, Physical Education and Recreation, Faculty of Science and Technology Education, University of Cape Coast, Cape Coast, Ghana

**Keywords:** Cervical cancer, HIV-positive, Women, Ghana, Developing countries

## Abstract

**Background:**

Cervical cancer affects women, especially those with HIV-positive status. This study hypothesised that more HIV-positive women with high cues about cervical cancer screening, high perceived susceptibility to cervical cancer, high perceived seriousness of cervical cancer, high perceived benefits of cervical cancer screening, and low perceived barriers about cervical cancer screening have intention to seek cervical cancer screening than HIV-positive women with low cues, low perceived susceptibility, low perceived seriousness, low perceived benefits, and high perceived barriers.

**Methods:**

A descriptive cross-sectional study was conducted with 660 HIV-positive women aged 20 to 65 years using an interviewer administered questionnaire. Data were summarised using frequencies, percentages and binary logistic regression analysis.

**Results:**

The findings showed that 82% (*n* = 540) of the respondents had intention to seek cervical cancer screening. The determinants of cervical cancer screening intention by HIV-positive women were cues, perceived seriousness and perceived benefits. Specifically, HIV-positive women with high cues were 3.48 times more likely to have intention to screen than those with low cues (95% CI, 1.43–8.49). Those with high perceived seriousness were 2.02 times more likely to have intention to screen than those with low perceived seriousness (95% CI, 1.24–3.30). Similarly, those with high perceived benefits were 1.7 times more likely to have intention to screen than those with low perceived benefits (95% CI, 1.05–2.71). However, perceived susceptibility (*p* = 0.063, OR 2.57, [95% CI, 0.95–6.93]) and perceived barriers (*p* = 0.969, OR = 1.01, [95% CI, 0.54–1.88]) were not statistically significant predictors of intention to seek cervical cancer screening in the sample studied.

**Conclusions:**

Cervical cancer screening interventions for HIV-positive women need to have a strong focus on explaining the seriousness of the disease, benefits of screening, and increase cues about screening, as these factors could improve attitude towards cervical cancer screening and promote the health of high risk women.

## Background

Globally, cervical cancer is one of the major causes of morbidity and mortality among women, especially those in Africa. In sub-Saharan Africa, over 80% of cancer of the cervix cases is detected at the terminal stages because of inadequate disease information and facilities for early detection [1, 2]. HIV-positive women are known to be at high risk of cervical cancer due to immunosuppression that is associated with HIV infection [3]. Cervical cancer is mainly caused by the Human Papilloma Virus (HPV). Several studies have reported a strong relationship between human immunodeficiency virus (HIV), HPV and cervical cancer [4–6]. A systematic review of HIV and cervical cancer in sub-Saharan African populations showed a strong association between HIV infection and neoplasia [7]. Ndiaye et al. and Holmes et al. found a correlation between HIV, HPV types 18 and 45 and cervical cancer [6, 8]. Despite the increased level of susceptibility to cervical cancer by HIV-positive women, data regarding HIV-positive women with cervical cancer in Ghana are not available due to the absence of a national cancer registry. The two hospital-based cancer registries in Ghana are in Korle-Bu Teaching Hospital, Accra and Komfo Anokye Teaching Hospital in Kumasi. These cancer registries are limited as they only capture cancer cases that report to these facilities.

In addition, although there is high incidence and mortality rates of cervical cancer in sub-Saharan cultures, screening for early detection of precancerous lesions are not frequently done. Meanwhile, HIV-positive women should screen for cervical cancer due to the high prevalence that has been observed, and the faster advancement of cervical precancerous lesions among this population [9].

Nonetheless screening, even once, could potentially minimise cervical cancer mortality among HIV-positive women in Africa [9]. Cervical cancer screening could detect HIV-positive women who might be at risk for developing invasive cervical cancer. Therefore early detection is recommended in ensuring regular follow up and effective treatment [10].

Previous studies have examined cervical cancer screening behaviour of college students, perceptions of men and women towards cervical cancer screening [1, 11–13]. Although HIV-positive women are more vulnerable to cervical cancer, there is a paucity of data on the factors influencing intention to obtain cervical cancer screening by HIV-positive women in the Central Region of Ghana to date. Therefore, the study hypothesised that more HIV-positive women with high cues about cervical cancer screening, high perceived susceptibility to cervical cancer, high perceived seriousness of cervical cancer, high perceived benefits of cervical cancer screening, and low perceived barriers about cervical cancer screening have intention to seek cervical cancer screening than HIV-positive women with low cues, low perceived susceptibility, low perceived seriousness, low perceived benefits, and high perceived barriers.

## Methods

A descriptive cross-sectional survey design was employed to study HIV-positive women in the Central Region, which is in the southern part of Ghana. The region has been associated with high rates of teenage pregnancy, HIV and Acquired Immunodeficiency Syndrome (AIDS) [14]. In 2014, the Central Region recorded HIV prevalence of 1.4% which was slightly higher than the national average of 1.37 in the 2014 HIV Sentinel Survey [15]. The study was conducted among HIV-positive women receiving antiretroviral therapy in health care facilities in the Central Region of Ghana. The Health Information Unit of the Central Regional Health Directorate suggested that a total of 3483 women between the ages of 20 to 65 years of age were receiving HIV/AIDS care [16].

### Sample size determination and sampling method

In determining the sample size the following conditions were considered: the research design was a cross-sectional survey and therefore required a larger sample size. Confidence levels were estimated at 95%, confidence interval or margin of error of plus or minus 4%, and variability probability of 50%. The 50% variability probability was based on the assumption that the population will split 50/50 on the question [17]. Based on these parameters, a sample size of 600 was needed. This was increased to 660 as a larger sample size reduces the degree of uncertainty [18]. Simple random sampling was used to select six of eleven health facilities responsible for providing care for people living with HIV in the Central Region to participate in this study. They were: Cape Coast Teaching Hospital, Abura Dunkwa Hospital, Swedru Government Hospital, Our Lady of Grace, Assikuma, St Francis Xavier, Assin Foso, and Saltpond Government Hospital. In order to obtain the required number of participants from each facility, the probability proportionate to size sampling was used to determine the proportions from each facility to be included in the sample. Accidental quota sampling was used to obtain the number of participants from each participating facility. Therefore, HIV-positive women within 20 and 65 years were selected until the desired sample size was reached.

### Data collection

A questionnaire was used to collect relevant data for the study. This was adapted from Mupepi et al. and Hassani et al. [5, 19]. Selection of measures was guided by the Health Belief Model (HBM) and Theory of Planned Behaviour (TPB) [20, 21]. These theories attempt to explain the category of people who will take appropriate action to prevent disease and predict their likelihood or intention towards preventive health-related behaviour. Measures based on these models have been used extensively in assessing cervical cancer screening behaviour among healthy as well as disadvantaged and marginalised populations [5, 22–24].

The measures for perceived susceptibility, perceived seriousness, perceived benefits, cues (prompts, triggers or reminders about cervical cancer screening) influencing cervical cancer screening, and perceived barriers were guided by the HBM. These constructs were measured on a four point-Likert scale. The use of a four point Likert scale is highly justified. Garland explained that social desirability bias, a situation where respondents give answers that put them in good light can be reduced by deleting or eliminating the mid-point from Likert scales [25]. Therefore, the responses to the Likert scale items were: strongly agree (SA), agree (A), disagree (D), and strongly disagree (SD). For positive statements, (SA) had a score of 4, (A) = 3, (D) = 2 and (SD) = 1. The reverse score was used for negative statements.

The details of questions used to assess the different parameters that were assessed have been described. For perceived susceptibility, the questions assessed were I worry about developing cervical cancer; I had a relative with cancer, so I may get cervical cancer; I don’t think I can get cervical cancer; I had multiple sexual partners in the past, so I may get cervical cancer; I have been in a polygamous relationship in the past, so I may get cervical cancer; I may not get cervical cancer because I already have HIV; I think I may get cervical cancer sometime in my life; and I do not feel at risk of getting cervical cancer. The items used to measure perceived seriousness included cervical cancer makes life worse; cervical cancer makes it difficult to have sex; cervical cancer patients may die within a short time; cervical cancer is more serious than other diseases; the problems caused by cervical cancer remain for a long time; cervical cancer makes patients anxious all the time; many women with cervical cancer may have complications; and cervical cancer mainly affects sexually active women.

In the same way, perceived benefits was measured with the following items: screening can find cervical changes before they become cancer; if cervical changes are found early they are easily treatable; screening test will help a woman to know if she has cervical cancer; screening cannot prevent the spread of cervical cancer; screening cannot save the patient’s life; cervical cancer screening may cause infertility; and if I had cervical cancer screening, I would never get cancer. The items on the perceived barriers to cervical cancer screening subscale comprised: I know where to go for cervical cancer screening; I don’t have much information about cervical cancer; the screening centres are too far from where I live; cervical cancer screening would be embarrassing; cervical cancer screening would be painful; cervical cancer screening is against my religious beliefs; I am afraid of knowing that I have cancer; and I cannot afford the cost of screening.

The items that constituted cues about cervical cancer screening were: education on the need for cervical cancer screening at the hospital will encourage me to have the test; If a relative suffers from cervical cancer, it will discourage me from obtaining the screening; If I hear about cervical cancer screening on the radio and television, I will go for the test; If I am referred for cervical cancer screening by my doctor, I will obtain the test; and If a doctor/nurse reminds me, I will obtain the test.

The instrument was shown to experts in the area of cervical cancer to judge it against the purpose and hypotheses the study sought to answer. To achieve internal reliability, a pilot study was conducted to ensure that the questions were appropriate and would be understood by the study participants. The pilot-test was conducted among 100 HIV-positive women in Effia Nkwanta Hospital, Takoradi, Western Region of Ghana. The pilot study was useful in identifying the type of training the data collectors might require before embarking on the main study [18]. Preliminary analysis was conducted to ensure that the data collected would answer the hypotheses. The Cronbach’s alpha internal consistency indices were obtained for the subscales were .824 for perceived susceptibility, .820 for perceived seriousness, .798 for perceived benefits, .809 for cues about cervical cancer screening and .795 for perceived barriers.

Ethical approval for the study was obtained from the Institutional Review Board of the University of Cape Coast and Ethical Review Committee of the Ghana Health Service. Approval was also obtained from the Ethical Review Committee of the Cape Coast Teaching Hospital. Permission was also sought from the Central Regional Health Directorate to use all the HIV/AIDS clinics randomly selected for the study in the Central Region of Ghana. Written informed consent was obtained from the participants before embarking on the data collection.

Participants were recruited at the hospitals, health centres and by word of mouth by professional nurses who had been trained in the care and management of HIV/AIDS patients. The benefit of participating in the research process was explained to the participants without providing misleading information or exaggerating about potential benefits. An interviewer-administered questionnaire was used. The independent variables for the study were; perceived susceptibility, perceived seriousness, perceived benefits, perceived barriers, and cues about cervical cancer screening. The dependent variable was intention to obtain cervical cancer screening. HIV-positive women who met the inclusion criteria were approached and interviewed after obtaining their full consent. Six nurses who could speak both English and Fante or Twi (native languages spoken by most people in the Central Region of Ghana) and had worked or were currently working with women with HIV were trained to assist with the data collection. The data were collected on special clinic days designated for caring for people with HIV/AIDS in the hospitals selected for the study. The interviews took place in the counseling rooms in the HIV/AIDS clinics from March to May, 2016. The data were analysed using frequencies, percentages and binary logistic regression. Respondents with a score of less than 75% for cues, perceived seriousness, perceived benefits, perceived susceptibility, and perceived barriers were considered to have low perception about the construct being measured while those with scores of 75% or more had high perception [26].

## Results

The socio-demographic characteristics of the respondents showed that, 89.7% (*n* = 592), of HIV-positive women were Christians while 10.3% (*n* = 68) were Muslims. In terms of the age distribution, 20.8% (*n* = 137) were between 20 and 29 years, 24.7% (*n* = 163) were within 30–39 years, 26.7% (*n* = 176) were within 40–49 years, 18% (*n* = 119) were within 50–59 years, and 9.8% (*n* = 65) were within 60–65 years of age. Regarding marital status, 40.5% (*n* = 267) were married, 24.2% (*n* = 160) were widowed, 14.1% (*n* = 93) were divorced, 12.7% (*n* = 84) were cohabiting, and 8.5% (*n* = 56) were single/had never married. Again, 21.7% (*n* = 143) had no formal education, 21.5% (*n* = 142) had primary education, 40.6% (*n* = 268) had attained secondary education, and 16.2% (*n* = 107) had attained tertiary education. In relation to the employment status of the respondents, 54.2% (*n* = 358) were employed, 40.6% (n = 268) were unemployed, 3.0% (*n* = 20) were students, and 2.1% (n = 14) had retired. In addition, 55.3% (*n* = 365) perceived the cost of cervical cancer screening as not affordable, 24.8% (*n* = 164) found it to be fairly affordable while 19.8% (*n* = 131) had the perception that it was affordable.

Out of the 660 HIV-positive women who participated in the study, 82% (*n* = 540) had intention to seek cervical cancer screening while 18% (*n* = 120) had no intention of participating in cervical cancer screening. Table 1 shows the classification of respondents’ responses into low and high groups on the basis of perceived susceptibility, perceived seriousness, perceived benefits, perceived barriers, and cues. From Tables 1, 86% (*n* = 573) had low susceptibility perception. Regarding the respondent’s perception of the seriousness of cervical cancer, Table 1 shows that 46% (*n* = 305) had high perception of the seriousness of cervical cancer. Similarly, 53% (*n* = 352) of the respondents had high perception of the benefits of cervical cancer screening.

**Table 1:** Summary of Descriptives on Perceived Scales

Perceived Scales	Frequency	Percentage
Perceived Susceptibility		
High	87	13.2
Low	573	86.8
Perceived Seriousness		
High	305	46.2
Low	355	53.8
Perceived Benefits		
High	308	46.7
Low	352	53.3
Perceived Barriers		
High	148	22.4
Low	512	77.6
Cues		
High	130	19.7
Low	530	80.3
Total	660	100.0

*N* = 660

The binary logistic regression analysis in Table 2 showed that cues about cervical cancer screening significantly predicted intention to screen (*p* = 0.006). Using respondents in the low group as reference category for the various scales, those with high cues about screening were 3 times more likely to have intention to screen (OR = 3.48, [95% CI, 1.43–8.49]). Table 2 shows that perceived seriousness significantly contributed to the model with a *p*-value of (*p* = 0.005). Respondents with high perception of the seriousness of cervical cancer were 2 times more likely to have intention to screen (OR = 2.02, [95% CI, 1.24–3.30]). Perceived benefits contributed significantly to the model with a *p*-value of (*p* = 0.032). Table 2 showed that respondents with high perceived benefits of cervical cancer screening were approximately 2 times more likely to have intention to screen (OR = 1.68, [95% CI, 1.05–2.71]). However, perceived barriers (*p* = 0.969, OR = 1.01, [95% CI, 0.54–1.88]) and perceived susceptibility (*p* = 0.063, OR 2.57, [95% CI, 0.95–6.93]) did not statistically significantly contribute to the prediction of intention to screen for cervical cancer.

**Table 2:** Binary Logistic Regression to Predict Respondents’ Intention to Seek Cervical Cancer Screening based on Perceived Scales

Variables	n (%)	*Β*	Wald	*p*-value	OR	95% CI for OR	
						Lower	Upper
Cues							
High	124(23)	1.25	7.53	0.006	3.48	1.43	8.49
Low (Ref.)	416(77)						
Perceived Seriousness							
High	272(50)	0.71	7.96	0.005	2.02	1.24	3.30
Low (Ref.)	268(50)						
Perceived Benefits							
High	269(50)	0.52	4.59	0.032	1.68	1.05	2.71
Low (Ref.)	271(50)						
Perceived Barriers							
High	127(24)	0.01	0.01	0.969	1.01	0.54	1.88
Low (Ref.)	412(76)						
Perceived Susceptibility							
High	82(15)	0.94	3.46	0.063	2.57	0.95	6.93
Low (Ref.)	458(85)						
Constant		2.16	35.35	0.001	8.70		

## Discussion

An important finding of this study is that high cue was a determinant of intention to seek cervical cancer screening by HIV-positive women. A possible explanation may be that high cues will enable HIV-positive women to have adequate information about screening which may influence screening behaviour. For instance, it may enable them to know where to go for the test, what the test entails and the benefits derived from screening. Previous studies found high cues to be a strong predictor of cervical cancer screening intention and actual screening [12, 27–32]. These studies pointed out that doctors’ recommendation, invitation letters, advertisements about the disease, and access to health education about the disease in primary care facilities would increase cervical cancer screening intention and utilisation among women, especially those living with HIV. In South Africa, HIV-positive women engaged in cervical cancer screening because medical doctors provided them with that important information [30]. Access to primary health care seems to influence intention as women who had such services had higher chances of having intention to seek screening compared with those with limited access [30, 31].

This finding implied that HIV-positive women with low cues may be at high risk of developing the disease. These women may not have adequate information about the disease to empower them to seek cervical cancer screening. Earlier empirical works have also emphasised the need for some cues to enable women to engage in cervical cancer screening [11, 33]. Interventions to increase cues about cervical cancer screening should be targeted to reach this HIV-positive group.

In addition, the findings showed that perceived seriousness of cervical cancer was a determinant of cervical cancer screening intention. A possible explanation for this outcome could be that HIV-positive women with high seriousness perception may be knowledgeable about the fact that cervical cancer is an equally devastating and deadly disease. Previous studies found perception of seriousness of cervical cancer to have predicted cervical cancer screening behaviour [31, 34, 35]. The consistency of the present study with previous ones could be due to the similarities in research design and theoretical basis that guided the studies. Thus, operationalisation of concepts would have been similar across the studies. For instance, the study by Ho et al. was conducted among Vietamese immigrants residing in the United States but the items used to measure perceived seriousness of the disease were similar to that of the present study. In the same way, the study by Ncube et al. was conducted among Jamaican women and knowledge about the consequences of cervical cancer led to cervical cancer screening as cervical cancer was perceived to have dire consequences on those suffering from the disease. If cervical cancer is not detected early through screening and managed, could have serious complications on those affected with the disease [36–38]. This makes it imperative for HIV-positive women to avail for screening for any cervical abnormalities to be detected before cancer develops.

Furthermore, perceived benefits predicted intention to screen as respondents had knowledge about the benefits of screening. In the current study, 52% of the respondents strongly agreed and 46% agreed to the statement that screening test will help a woman to know if she has cervical cancer. Similarly, 48% strongly agreed and 46% agreed to the statement that screening can find cervical changes before they become cancer. The result of this study is consistent with evidence from previous studies conducted in Israel and Poland [39, 40].

A meta-analysis concluded that cervical cancer screening offers substantial protective benefits to women, especially when screening is done for women aged 30 years and above [41]. Although evidence from meta-analysis are considered to be of high quality and can inform practice, the studies included in the review were randomised control trials and observational studies conducted in advanced settings. Nonetheless, the finding is of relevance to developing settings as few studies that have been conducted in resource poor settings have drawn similar conclusions [5, 42]. It seems in settings where cervical cancer screening programmes are well established, awareness and knowledge about the benefits may positively influence screening intention. Women who engage in cervical cancer screening tend to have control over their life, which can result in high performance in other aspects of their life [43].

Perceived barrier was not found to be a determinant of intention to screen. This is consistent with previous findings [5, 12]. It is well documented that potential barriers could be in the form of institutional factors, personal, negative belief, social, fatalistic, financial, and negative misconception, which tends to hinder women’s ability to engage in cervical cancer screening. In the same way, perception of susceptibility did not determine intention to seek cervical cancer screening [5, 12]. This finding implied the need for HIV-positive women to be educated on the risk factors for cervical cancer such as having multiple sexual partners as well as the complications associated with the disease. It seems they may not be aware that they are highly susceptible to the disease.

## Conclusions

The findings of this study suggested that high perceived seriousness of cervical cancer, high cues and high perceived benefits of cervical cancer screening were the most important factors that determined intention to seek cervical cancer screening by HIV-positive women. It further demonstrated that HIV-positive women with low cues, low perceived seriousness of the disease and low perceived benefits may not engage in cervical cancer screening. The finding has an important implication for public health education on cervical cancer and screening for HIV-positive women. Health education programme on cervical cancer and screening should be designed to enable HIV-positive women access the essential information about the disease and screening. It could be deduced from the findings of the current study that when HIV-positive women are educated about cervical cancer screening including the seriousness and benefits of participating in screening activities, they may have better chances of utilising screening services. It is critical that cues about screening are intensified to facilitate access to essential information. Nonetheless, it is evident that HIV-positive women may have barriers that could hinder intention to screen and subsequent attempt to utilise cervical cancer screening. This highlights the fact that the perceived barrier construct in the HBM actually impedes intention to screen. Despite evidence that perceived susceptibility influences intention to screen, the current study proposes that perceived susceptibility may not commit HIV-positive women to have intention to screen. This implies that education on cervical cancer screening for HIV-positive women needs not to strongly focus on their level of perceived susceptibility since it may not be a crucial factor in determining intention to screen.

## Abbreviations

A: Agree
AIDS: Acquired Immunodeficiency Syndrome
D: Disagree
HBM: Health Belief Model
HIV: Human Immunodeficiency Virus
HPV: Human Papilloma Virus
SA: Strongly Agree
SD: Strongly Disagree
TPB: Theory of Planned Behaviour
